# Aseismic mid-crustal magma reservoir at Cleveland Volcano imaged through novel receiver function analyses

**DOI:** 10.1038/s41598-020-58589-0

**Published:** 2020-02-04

**Authors:** Helen A. Janiszewski, Lara S. Wagner, Diana C. Roman

**Affiliations:** 1 Department of Terrestrial Magnetism, Carnegie Institution for Science, Washington, DC, United States; 2 Present Address: Department of Earth Sciences, University of Hawai‘i at Mānoa, Honolulu, HI United States

**Keywords:** Seismology, Volcanology

## Abstract

Processes related to eruptions at arc volcanoes are linked by structures that transect the entire crust. Imaging the mid- to lower-crustal portions (here, ~5–15 km and >15 km respectively) of these magmatic systems where intermediate storage may occur has been a longstanding challenge. Tomography, local seismic source studies, geodetic, and geochemical constraints, are typically most sensitive to shallow (<5 km) storage and/or have insufficient resolution at these depths. Geophysical methods are even further limited at frequently-erupting volcanoes where well-developed trans-crustal magmatic systems are likely to exist, due to a lack of deep seismicity. Here we show direct evidence for mid-crustal magma storage beneath the frequently erupting Cleveland volcano, Alaska, using a novel application of seismic receiver functions. We use P-s scattered waves from the Moho as virtual sources to investigate S-wave velocities between the Moho and the surface. Our forward modeling approach allows us to provide direct constraints on the geometry of low velocity regions beneath volcanoes despite having a comparatively sparse seismic network. Our results show clear evidence of mid-crustal magma storage beneath the depths of located volcanic seismicity. Future work using similar approaches will enable an unprecedented comparative examination of magmatic systems beneath sparsely instrumented volcanoes globally.

## Introduction

Recent research on trans-crustal magmatic systems (TCMS) demonstrates the importance of relationships between temporally longer-lived zones of crystals, melt, and volatiles (“mush”) that extend throughout the crust, and comparatively ephemeral and depth-restricted magma reservoirs comprised primarily of eruptible melt^1^ in driving volcanic eruptions. A longstanding barrier to understanding these systems is the challenge of imaging the mid- to lower-crustal portions of TCMS^1,2^. Commonly-used approaches for detecting crustal magma reservoirs include analyses of volcanic seismicity, analyses of geodetic observations, and geochemical and petrological analyses of eruption products. Volcanic seismicity is linked to differential strain and pressure transfers related to magma and volatile movement in the crust and can either be similar to tectonic earthquakes (volcano-tectonic or VT events) or have primarily long period energy (LP events)^3–7^. Similarly, geodetic methods constrain locations of magma storage by measuring deformation associated with magma emplacement and movement within the crust, and are typically most sensitive to shallow magma storage regions^8–10^. Both methods are most sensitive to the results of magma movement; therefore, the absence of seismicity or geodetic deformation at volcanoes cannot be interpreted as the absence of stable magmatic (or mush) storage regions, limiting the ability of these techniques to provide information on comparatively stable volcanic systems. Geochemical and petrological approaches for assessing magma storage depths are most sensitive to the region of last storage in the crust prior to eruption^11^. In most volcanic arcs, the depth of final equilibration is located in the uppermost ten kilometers of crust^12^, obscuring the storage history in the mid- and lower-crust. These techniques also limit samples to that of erupted magma, ignoring the majority that is emplaced in the crust^13,14^.

Local and active seismic tomography are useful in characterizing crustal magma storage beneath volcanoes, as they are not necessarily tied to changes in stress or pressure related to crustal magma transport, or access to samples of erupted products. Recent results from high-density seismic deployments have revealed complex three-dimensional magma storage regions with scales ranging from the upper few kilometers to the whole crust^15–19^. While there is promise for reliable local seismic tomography with modest numbers of instruments, these studies are still limited in their ability to image the full TCMS by the availability of local seismicity at mid–to–lower crustal depths^20–22^. Recent developments in ambient noise tomography also show promise in imaging large magmatic complexes, however are limited by their resolution for imaging at smaller, individual volcanic systems^23,24^.

Here, we circumvent the limitations of local earthquake tomography by using P-to-s conversions of teleseismic earthquakes at the Moho, identified using receiver functions, as virtual sources to image the velocity structure of the lower crust beneath Cleveland volcano, Alaska. We present evidence of a narrow and vertically-extensive TCMS in the lower crust beneath this highly active open-system volcano. Specifically, our results require the presence of a low velocity zone with a minimum vertical extent of 10–17.5 km depth below sea level with a relatively small diameter <5 km. Furthermore, our novel approach now permits first-order seismic imaging of relatively small-scale features at sparsely-instrumented (<~10 seismometers) volcanoes worldwide.

## Receiver Functions as Synthetic Sources

The ability to constrain crustal thickness and velocity structure beneath individual seismic stations primarily comes from the identification of conversions of teleseismic P-waves to S-waves at the Moho using receiver functions^25^. The difference in arrival time between the direct P-wave and the P-s Moho-converted phase can be migrated to depth if the crustal velocity structure is known. It is not uncommon to assume a laterally homogeneous crustal structure when performing such migrations, especially in the absence of independent constraints such as Rayleigh wave phase velocities^26–29^. However, in the presence of strong abrupt changes in seismic velocities within the crust, the apparent crustal thickness determined using a laterally homogeneous velocity model will become inconsistent as a function of Moho pierce point depending on the travel path of the converted wave^30,31^. It is precisely such an observation that has led us to use the differential travel time between the direct P-wave and the P-s converted wave beneath Cleveland volcano to examine crustal velocity structure. This technique circumvents the lack of deep seismicity that has been a significant limiting factor in the ability to use seismology to image mid–to–lower crustal structure beneath many volcanoes.

**Figure 1:**
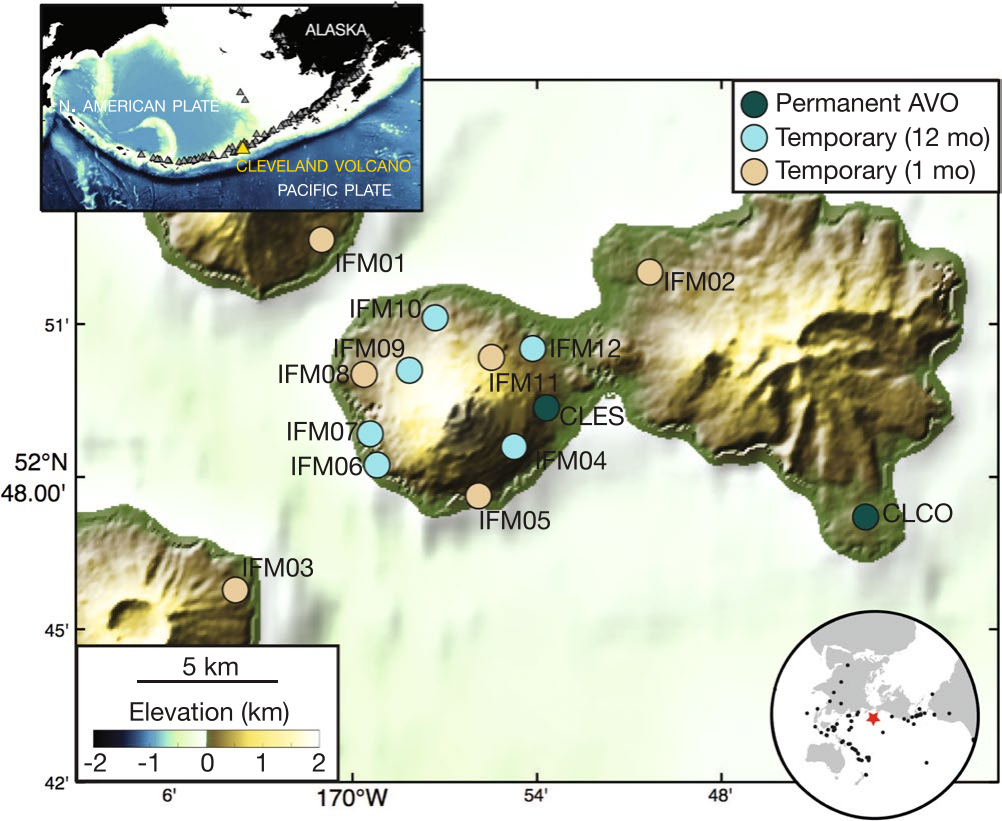
Seismic station map. Dark blue circles are permanent Alaska Volcano Observatory (AVO) broadband seismic stations operating since 2014. Light blue are broadband stations that operated for twelve months. Tan are broadband stations that operated for one month. Top inset: Location of study region in the Aleutian island arc. Bottom inset: Locations of teleseismic earthquakes used for receiver functions (black dots) relative to study area (red star). Earthquake information is given in Table S1. The software package GMT^57^ (version 5.4; generic-mapping-tools.org) has been used to produce the figure using GMRT^58^ topography data.

Data for this study were recorded by eight broadband seismometers deployed around Cleveland volcano that operated for at least a year (Fig. 1). Six of these stations were deployed as part of the NSF-funded Islands of the Four Mountains experiment between August 2015–July 2016^32^. In addition, we analyze data from August 2014 through September 2018 from two permanent Alaska Volcano Observatory (AVO) stations deployed at Cleveland volcano. We calculate receiver functions from both teleseismic *P* and *PP* arrivals recorded at all stations in the network (Fig. 1).

### Moho depth versus crustal velocity

**Figure 2:**
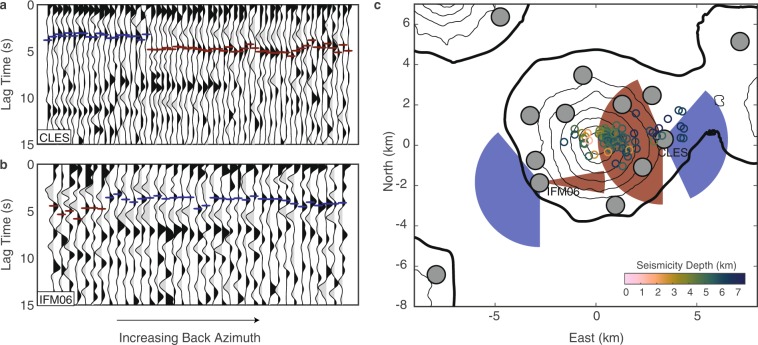
Example receiver functions. (**a**) Station CLES (top) located to the east of the Cleveland edifice. (**b**) Station IFM06 (bottom) located to the west of the edifice. For both, black indicates positive arrivals, grey indicates negative. Red and blue lines indicate picked Moho *Ps* arrivals for late and early lag times, respectively. (**c**) Back azimuthal variation in the picked arrival times. Gray circles indicate seismometers, with CLES and IFM06 labeled. Red and blue shaded regions indicate the back azimuthal ranges for the late and early arrivals at left; late arrivals (red) consistently point back towards the volcanic edifice, and early arrivals point away. Small circles indicate local VT seismicity colored by depth below sea level^40^. The software package M_Map^59^ (version 1.4; www.eoas.ubc.ca/~rich/map.html) has been used to produce the figure using GMRT^58^ topography data.

Receiver functions around Cleveland volcano show clear arrivals of crustal phases within the first 6 s that we interpret as P-s conversions from the Moho. However, we observe variations in the converted Moho arrival times of up to ~2 seconds depending on the path of the P-s converted wave between the Moho and the station (Fig. 2).

**Figure 3:**
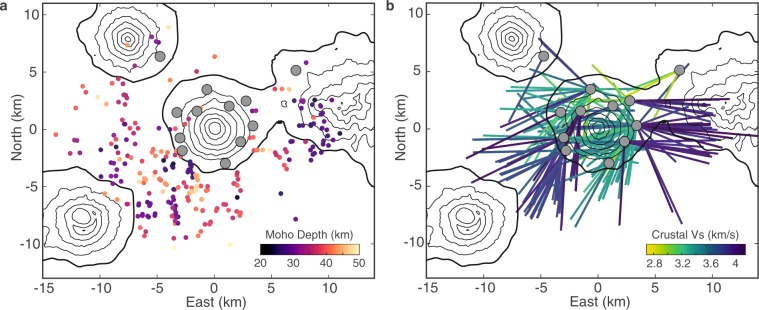
Comparison between two end-member models for *Ps* lag time. (**a**) Variation in Moho depth needed to explain *Ps* variation. Colored dots show the Moho depth estimated for each receiver function, plotted at the pierce point of the ray path through the Moho. (**b**) Variation in average crustal shear velocity needed to explain *Ps* variation. Colored lines show the estimated crustal *Vs* for each receiver function, plotted along its crustal ray path. Grey circles indicate seismometers. The software package M_Map^59^ (version 1.4; www.eoas.ubc.ca/~rich/map.html) has been used to produce the figure using GMRT^58^ topography data.

To investigate the cause of this arrival time variation, we determine the difference between the direct P-wave arrival time and the P-s converted wave arrival time to calculate the travel time of the S-wave through the crust. We then calculate a) the depth to the scattering discontinuity (varying depth to Moho) assuming a fixed crustal seismic shear velocity (3.7 km/sec) and b) the average seismic velocity between the pierce point of the converted S-wave and the station assuming a fixed crustal thickness (30 km) (Fig. 3). Prior results suggest an average crustal P-wave velocity of 6.5 km/s^33^, and S-wave velocity of 3.7 km/s, or *Vp/Vs* of 1.76^34–36^. We assume a Moho depth of 30 km, based on a best-fit to our *Ps* Moho conversions for ray paths away from the volcanic edifice. This Moho depth is shallower than previous results averaged for the whole Aleutian arc (~35 km^34^), possibly indicative of small-scale variability in Moho depth in the Aleutians.

If crustal velocities are kept constant, the range in crustal thicknesses required by the variable arrival times would need to vary between 20 and 50 km depth beneath the 20 km × 20 km region surrounding Cleveland Volcano (Fig. 3a). Given the proximity of pierce points with different scattered wave arrival times, crustal thicknesses would be required to vary by as much as 20 km over distances of less than 5 km horizontally. While Moho depth variability on the order of 20 km is observed in other volcanic regions^37^, it is over distances of hundreds of kilometers and is less spatially random than in our observations.

Alternatively, if the Moho depth is kept constant at an average depth of 30 km, shear wave velocities would need to vary from ~2.8 to 4 km/s (Fig. 3b) to explain arrival time variability. The distribution of velocities is bimodal; ray paths crossing the volcanic edifice are distributed around a shear velocity of ~3.3 km/s, while ray paths not crossing the edifice are centered around a shear velocity of ~3.9 km/s. This range in shear wave velocities is similar to velocities observed in other volcanic settings such as the Taupo volcanic region^38^, Iceland^37^, Mt. Aso^39^, and Akutan^34^. Therefore, while it is likely that both the crustal thickness and the shear wave velocities beneath Cleveland volcano vary, the dominant contributing factor to the offset in converted-wave arrival times must be variations in shear wave velocity.

### Depth extent of low crustal shear velocities

#### Forward modeling approach

**Figure 4:**
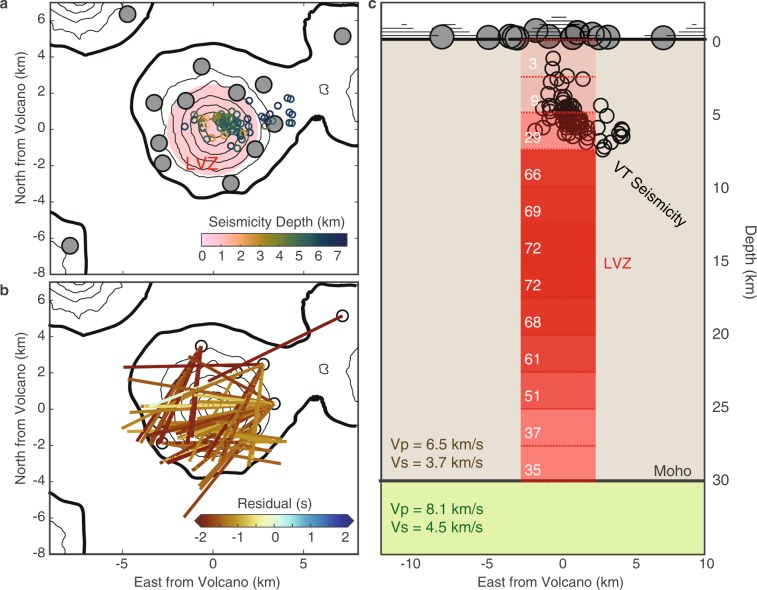
Forward model setup. (**a**) Location of cylindrical LVZ (red), VT seismicity (colored circles), and seismic stations (grey circles). (**b**) Residual *Ps* lag times for ray paths that pass through the region where the cylindrical LVZ is defined for the baseline model. Negative values indicate that the predicted lag time is earlier than the observed lag time. (**c**) Profile of the velocity model with baseline velocities for crust and Moho (beige and green regions), as well as the LVZ (red). Dashed red lines through the LVZ indicate 2.5 km intervals, and opacity indicates number of rays (indicated in white) that intersect the LVZ within each depth section. The software package M_Map^59^ (version 1.4; www.eoas.ubc.ca/~rich/map.html) has been used to produce the figure using GMRT^58^ topography data.

To constrain the vertical extent of the region of slow shear velocities observed for crustal ray paths crossing beneath the volcanic edifice, we calculate predicted travel times for converted waves through a series of velocity models with fixed crustal thickness that contain a cylindrical low velocity zone (LVZ) centered beneath the edifice. The radius of the cylinder is 2.5 km based on the lateral extent of the well-located volcano-tectonic (VT) earthquakes, which form a tight cluster beneath the edifice^40^ (Fig. 4a). A cylinder with a smaller radius does not explain the observations as well, while a larger radius significantly affects the ray paths away from the edifice that do not show evidence of slow crustal shear velocities (see Supplemental Information).

We allow the top and bottom of the LVZ to vary at intervals of 2.5 km through 30 km crust. All depths are defined below sea level. We then test three separate suites of LVZ models, each with different values of Vs within the LVZ (2 km/s, 2.5 km/s, and 3 km/s) in order to investigate trade-offs with LVZ height and location. Outside of the LVZ, Vs is fixed at 3.7 km/s, consistent with prior regional seismic studies^33–35^. New local seismic tomography results agree with these regional parameters, although they are primarily sensitive to the upper ~10 km of crust^41^. Uncertainties in the parameters used to describe the baseline crust do not significantly affect our analysis, since we focus primarily on the velocity differences between the baseline and the crust beneath the edifice. We only investigate the variation of *Vs* in the LVZ, leaving crustal *Vp* constant throughout our study region. P-to-s receiver functions are primarily sensitive to crustal shear velocity, and magmatic features typically affect *Vs* significantly more than *Vp*^21,42^, so only varying crustal *Vs* is a reasonable first order approximation. We define a baseline model that does not include an LVZ in the crust for comparison to the modeling results (Fig. 4b).

For each velocity model, we calculate the predicted lag time of converted *Ps* phase after the initial *P*-wave by ray tracing the converted wave from the station back to the pierce point of the Moho, and compare that to the observed lag time of the *Ps* phase. To focus only on those rays sampling the crust beneath the volcanic edifice, we calculate predicted lag times for the baseline model and for a full-crustal-thickness LVZ; only the 72 receiver functions with differing predicted lag times between these two models are included in subsequent calculations. This approach identifies the subset of ray paths that could be affected by the LVZ, allowing us to focus on structure in the region of seismicity. For each model test, this same set of receiver functions is used to evaluate misfit, even if some ray paths no longer cross the LVZ (e.g. pass above or below it when it is not the full crustal column). We define the misfit for each model as the root-mean-square (RMS) of the residual between the observed and predicted *Ps* lag time. The RMS misfit for the baseline model for this subset of receiver functions is 1.67 sec/ray. The RMS misfits for the full crustal column LVZ for 2 km/s, 2.5 km/s, and 3 km/s are 3.91 s/ray, 1.55 s/ray, and 0.74 s/ray respectively.

#### Mid- to deep-crustal low shear velocities

**Figure 5:**
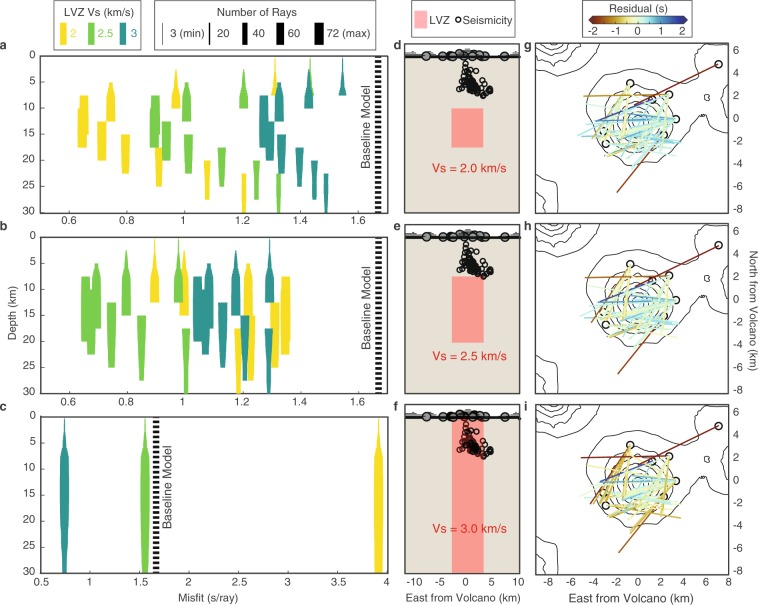
Modeling results for a columnar LVZ. (**a–c**) Misfits for models with an LVZ of a constant thickness (**a**), 7.5 km; (**b**) 12.5 km; (**c**) 30 km) for each depth range and *Vs* combination. The y-axis indicates the depth extent of the LVZ and x-axis indicates the average misfit for a particular model, bar color indicates *Vs*, and bar thickness indicates the number of rays that pass through the LVZ at a particular depth. The misfit for the baseline model is shown for comparison; note the change in misfit axis in the bottom plot. (**d–f**) Profile showing the best fit model for each of the three *Vs* values. (**g–i**) *Ps* lag time residuals for the best fit model (**d–f**) for all ray paths that intersect the LVZ at any depth. The software package M_Map^59^ (version 1.4; www.eoas.ubc.ca/~rich/map.html) has been used to produce the figure using GMRT^58^ topography data.

Figure 5 shows the RMS misfits of the models tested in this study. The best-fit models for the three shear velocity values tested indicate a region of significantly reduced velocities between, at a minimum, 10–17.5 km depth. Because there are trade-offs between Vs and depth, the different velocities indicate different, but overlapping, depth ranges for the best fitting LVZ. For *Vs* = 2.0 km/s, the best fit LVZ extends from 10–17.5 km depth with a misfit of 0.65 s/ray, for *Vs* = 2.5 km/s, it extends from 7.5–20 km depth with a misfit of 0.66 s/ray, and for *Vs* = 3 km/s it extends from 0–30 km depth with a misfit of 0.74 s/ray (Fig. 5). These misfits are improvements of 61%, 60%, and 56% respectively over the baseline model.

#### Model resolution

The sampling distribution of rays at different depths within the defined cylindrical LVZ provides insight into the resolution of our method. Figure 4c shows the possible number of rays sampling each 2.5 km depth bin for Vs = 3 km/s and assuming full crustal thickness. Only 29 of 72 rays sample the crust at or above the depth of the VT seismicity (~7.5 km), and there are almost no ray paths in the very shallow crust (<5 km). Therefore, improvements to the travel time misfit must be due, at least in part, to decreases in velocity below these depths. This result does not indicate the definitive absence of slow velocities directly beneath the edifice at shallow depths, but rather that our dataset is not sensitive to this region and a deeper source of slow velocities is required to explain the observations. Similarly, few rays pass through the deep crust (>25 km), although this is dependent on the velocity of the LVZ. Since the majority of the ray paths traverse the LVZ from 12.5–17.5 km depth (Fig. 4c), we expect the average residuals to be especially sensitive to velocity changes in this depth range, which is in line with the preferred depth range results (Fig. 5).

**Figure 6:**
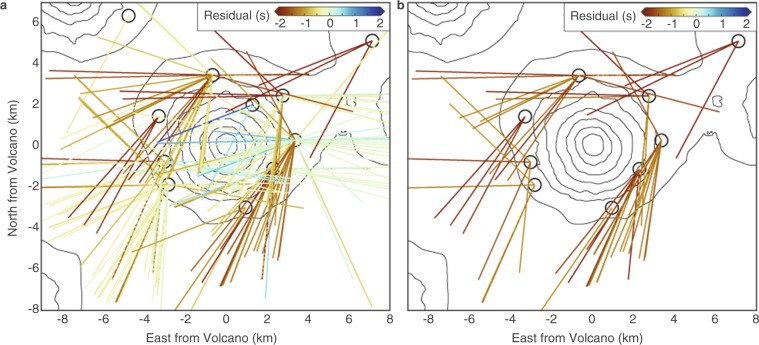
Model fit for all ray paths. (**a**) *Ps* lag time residuals for all ray paths with an LVZ extending from 7.5–20 km depth and *Vs* = 2.5 km/s. (**b**) *Ps* lag time residuals for the same model showing only ray paths with <−1 s residual. The software package M_Map^59^ (version 1.4; www.eoas.ubc.ca/~rich/map.html) has been used to produce the figure using GMRT^58^ topography data.

A simple cylindrical LVZ does not entirely account for all observations of anomalous *Ps* lag times in the receiver functions (Fig. 6a). Using the model with an LVZ of *Vs* = 2.5 km/s from 7.5–20 km as an example, the average misfit including all ray paths, not just those that pass under the edifice, is 0.90 s/ray. This is a 29% improvement from the baseline for all rays; ~40% of ray paths have residuals within 0.5 s of the observed lag times in the positive or negative direction while only ~30% have residuals <−1 s. The remaining ray paths with residuals <−1 s are not randomly distributed in space; they approximately form a perimeter around the volcanic edifice, with the exception of the southwest region of the island (Fig. 6b). This may point to an irregularly shaped crustal LVZ beneath Cleveland volcano, potentially elongated NW-SE, consistent with the regional crustal stress field in this segment of the arc^43^. Similar patterns exist for the best fit models for assumed *Vs* of 2 and 3 km/s. These observations indicate the potential for using receiver function analyses to constrain more irregularly-shaped velocity anomalies.

## Discussion

### Implications for mid-crustal magma storage

Our results demonstrate clearly that a low velocity zone beneath Cleveland volcano is required by the data, and that the most likely location of that LVZ is within the mid-crust (~10–20 km depth). The best fitting models have velocities of 2 or 2.5 km/sec, representing a decrease of 32– 46% over the defined average velocity outside of the LVZ. Given our tests, a shear velocity of 3 km/s can be considered an approximate maximum value for the LVZ since it requires a full crustal thickness column, which is still a 19% reduction in shear velocity. Following the approach used at other volcanic systems, the shear velocities of 2, 2.5, and 3 km/s are consistent with ~23, 16, and 8% melt fraction respectively^44^; however, relationships between seismic velocity and melt can be complicated by parameters such as melt connectivity and volatile content^45–48^. Nevertheless, these values are outside the range of global crustal shear velocities^49,50^, and are similar to observations interpreted as melt and/or fluids in other magmatic systems^17,39,51^. Therefore, we interpret the LVZ as a region of crustal magma storage beneath Cleveland volcano.

Furthermore, a qualitative examination of the receiver functions suggests a more vertically extensive storage region as opposed to a sharply bounded sill or lens-like structure. Receiver functions that pass under the edifice at Cleveland do not contain widespread evidence of additional arrivals distinct from the initial P-wave or Moho Ps conversion that would indicate mid-crustal discontinuities (see Supplemental Information). This lack of additional arrivals suggests that the mid-crustal LVZ is not defined by sharp horizontal boundaries at its top and base, and is instead more likely a vertically-elongated low-velocity zone beneath Cleveland volcano, indicative of a vertically extensive TCMS similar to what would expected for a “mush”-rich crust^1^. This implies that the low velocities do not represent recently injected dikes that have yet to thermally equilibrate with the surrounding host rock, and instead may indicate a longer-lived, more stable system. Cleveland volcano is one of the most active Aleutian volcanoes with frequent gas emissions, explosions and ash deposits^52,53^; however, with the exception of a brief swarm shallow seismicity recorded in 2015 there is no evidence of deformation. The TCMS extends significantly below all located VT seismicity, suggesting that it facilitates magma movement throughout the crust without significant differential strain or brittle failure that would lead to episodes of seismicity^4^, consistent with expectations for a well-developed open system volcanic conduit.

### Along-arc comparisons

This vertically extensive TCMS beneath Cleveland volcano significantly differs with observations from a similar dataset at nearby Akutan volcano^34^, where receiver functions traversing beneath the edifice contained a clear additional arrival prior to the Moho *Ps* conversion, while the travel time for the Moho *P-s* converted phase did not vary substantially. The Akutan observations are best explained with a relatively thin LVZ, ~1–3 km thick, with sharp boundaries at the top and base and located only between ~7–11 km depth beneath the entire island, consistent with laterally extensive and shallow VT seismicity^54^. Abundant deep LP seismicity is also recorded at Akutan^55^, and is interpreted as reflecting discrete episodes of magma transport rather than continuous flux through the deep crust^54^. Additional constraints on magmatic structure using traditional seismic tomographic techniques beneath Aleutian volcanoes include magma storage at ~7 km depth beneath Makushin^54^, and a TCMS at Mt. Spurr^42^. These examples, while not comprehensive, begin to demonstrate the variability in magmatic structures beneath volcanoes in a single volcanic arc motivating the need for future systematic arc-transect studies sensitive to the whole crust.

## Conclusions

Our technique of using *Ps* Moho conversions from receiver functions as virtual sources allows first-order imaging of the entire crust beneath sparsely-instrumented volcanoes. It is not dependent on the presence of deep seismicity, allowing imaging of relatively stable or dormant systems that do not currently have deep crustal activity. It is also able to image gradational seismic variation in the crust, as well as more abrupt sill like structures more traditionally imaged with receiver function techniques. This versatility and relatively low instrument investment of this technique allows for more flexibility in imaging many different types of volcanic settings, facilitating future comparative volcanology studies as opposed to traditional in-depth “lab-volcano” style experiments. Finally, we suggest that application of our approach to existing volcano-seismic data sets may help to guide the location and network design for more extensive seismic deployments aimed at full tomographic inversions for 3D velocity structure at these key “lab-volcanoes” worldwide.

## Methods

### Receiver function calculation

We calculate receiver functions from both teleseismic *P* and *PP* arrivals from earthquakes that occurred between 20–100° and 90–180° from Cleveland volcano respectively. For the temporary station data, we examine the *P* arrivals from earthquakes *Mw* ≥ 5.5 to maximize the number of earthquakes recorded during the deployment. For the permanent AVO stations we only examine *P* arrivals from earthquakes *Mw* ≥ 6.5. For all stations, we use *Mw* ≥ 6.5 for teleseismic *PP* arrivals. Seismic waveforms are visually inspected for teleseismic arrivals prior to calculating the receiver functions. Few earthquakes with *Mw* < 6.5 had clear arrivals on the temporary array; therefore, it is unlikely their inclusion would significantly improve back-azimuthal coverage at the two permanent stations.

We use the time iterative deconvolution method^56^ to calculate receiver functions. Prior to deconvolution the data are windowed from 10 seconds prior to and 90 seconds after the *P* or *PP* arrival, and high-pass filtered at 0.07 Hz. A low-pass (0.5 Hz) Gaussian filter is applied in the deconvolution. Receiver functions are visually inspected and those with a negative direct P-wave arrival (which should be positive) or an unclear Moho *Ps* arrival are discarded. After quality control, the permanent AVO stations provide ~60 receiver functions, while the temporary sites deployed for the full year provide ~30 receiver functions. Sites that operated for less than a month provide fewer than 5 receiver functions, but are still included in our analysis.

## Supplementary information

Supplementary Information.

Supplementary Information.
