# Supplementary material for: Aseismic mid-crustal magma reservoir at Cleveland Volcano imaged through novel receiver function analyses
Source: Sci Rep. 2020 Feb 4;10:1780. doi: 10.1038/s41598-020-58589-0 (PMC7000785; doi:10.1038/s41598-020-58589-0)
Supplement: Supplementary file 1 — Supplementary Information. [file 41598_2020_58589_MOESM1_ESM.pdf]

Aseismic mid-crustal magma reservoir at Cleveland Volcano imaged through novel receiver function analyses

Helen A Janiszewski[1,2 *], Lara S Wagner[1], Diana C Roman[1]

* corresponding author: hajanisz@hawaii.edu
[1.] Department of Terrestrial Magnetism, Carnegie Institution for Science
[2.] now at Department of Earth Sciences, University of Hawaii, Manoa

Supplementary Information

Receiver Function Observations

All good quality receiver functions are shown from Figures S1 – S13. These are from all of the stations that were deployed in the vicinity of Cleveland volcano, including six temporary stations that operated for a year, six temporary stations that operated for a month, and two permanent AVO stations that have operated since 2014. The stations that operated for only a month recorded less than four teleseismic earthquakes that were suitable for receiver function calculation, making examination of their back azimuthal patterns impossible, but are still included for completion. A table of the teleseisms used is included (Table S1).

The main feature present at all stations that recorded for at least a year with the exception of CLCO is a *Ps* arrival with a lag time that strongly varies with back azimuth, as discussed in the main text. For raypaths that predominantly pass beneath the volcanic edifice, the *Ps* arrival has a lag time of ~ 5 s; for ray paths that point away from the edifice, the arrival is typically ~ 3 s behind the initial *P* wave. We do not observe strong additional positive arrivals that are distinct from the initial P-wave arrival except at IFM09. At this station, there is an additional positive arrival at ~ 1 s lag time with higher amplitude than the initial P arrival at certain back azimuths. This is likely due to shallow crustal structure local to that station. This station still also exhibits the variation of *Ps* lag time seen across the array, but it is more difficult to interpret with the complex earlier arrivals. Therefore, we exclude it from the main forward modeling analysis. Despite the fact the CLCO is outside of the edifice region, the lag times for its *Ps* conversions are consistently ~ 5 s with no back azimuthal variation. However, its raypaths sample distinct crust, and do not cross with the raypaths recorded by the rest of the array. Thus, it is plausible that

the crustal structure beneath CLCO is different, with perhaps some combination of deeper Moho or slower average crustal velocities leading to the *Ps* lag times (Figure S14).

In addition, we observe consistent arrivals at lag times later than the *Ps* conversion, but they do not show similarly clear variations with back azimuth. These positive arrivals between 7 - 8 s lag time are most likely *Ps* conversions from the subducting oceanic Moho. They are also observed at station CLCO, so not likely a multiple from shallow structure local to the volcano. Positive arrivals observed at 10 - 12 s lag time are likely *Pps* Moho reflections. However, we do not consider these features further in this study.

Radius of Cylindrical LVZ

For our forward modeling approach, we chose a radius for the cylindrical LVZ that maximizes its extent beneath the edifice while not affecting the raypaths pointing away from the edifice that have shorter lag times for the *Ps* arrivals. This allows us to include a maximum number of rays that show evidence for slow velocities, while not affecting the raypaths that show no evidence of passing through an LVZ. The rationale behind this approach is essentially that we use a simplified cylindrical LVZ to focus on constraining the crustal structure in the vicinity of the main volcanic edifice and VT seismicity, with the recognition that an irregularly-shaped region is needed to explain all of the observations. We test several radii for the cylindrical LVZ to determine these approximate constraints on its diameter. Approximately one-third of the raypaths added by increasing the radius to 3 km from 2.5 km that traverse the LVZ region are well fit by the baseline model with no LVZ (residual < 0.5 s) (Figure S15). This indicates that a simple cylindrical LVZ cannot describe the raypaths that pass through it when

the radius is increased to 3 km from 2.5 km, and a more complex geometry will be needed to accurately determine the structure that can describe these raypaths.

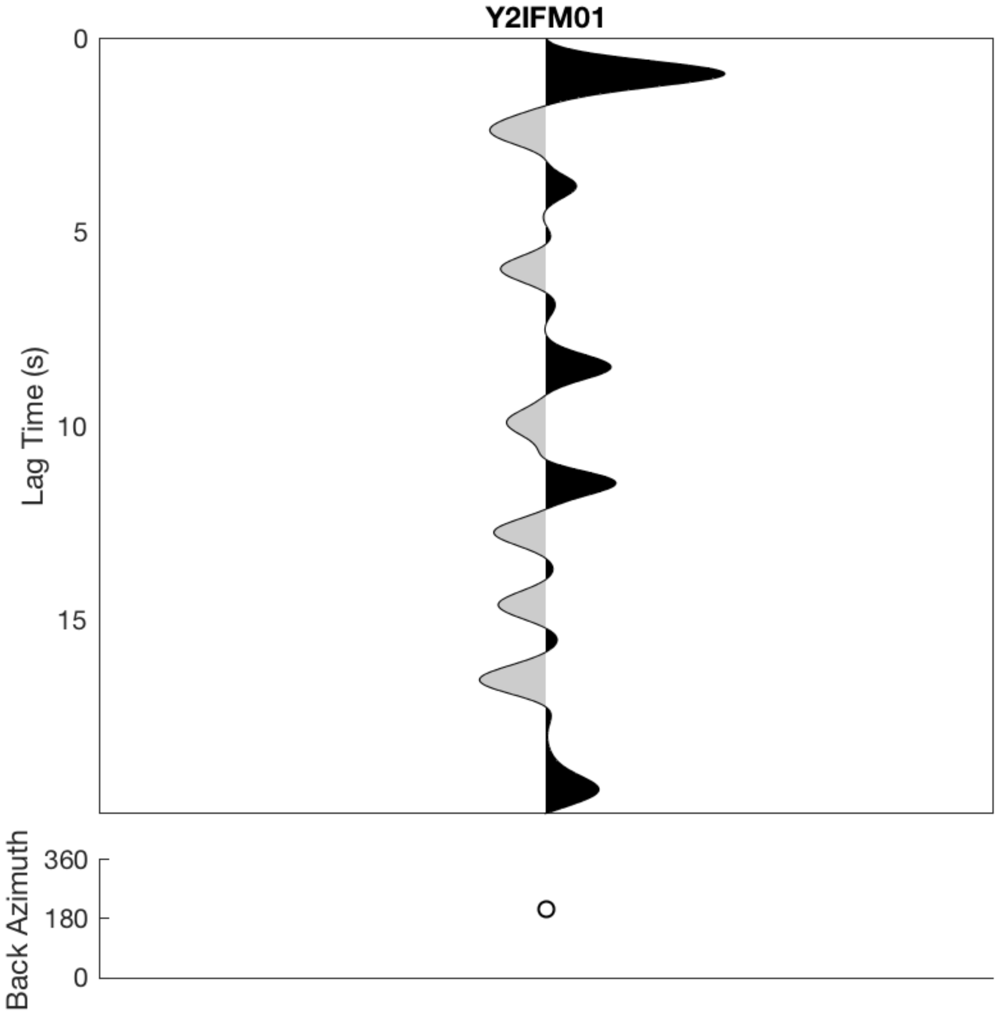

Figure S1: Receiver functions from station Y2IFM01. Black arrivals are positive, grey are negative. The receiver functions are shown in order of increasing back azimuth; the plot at the bottom shows the back azimuth for each individual receiver function.

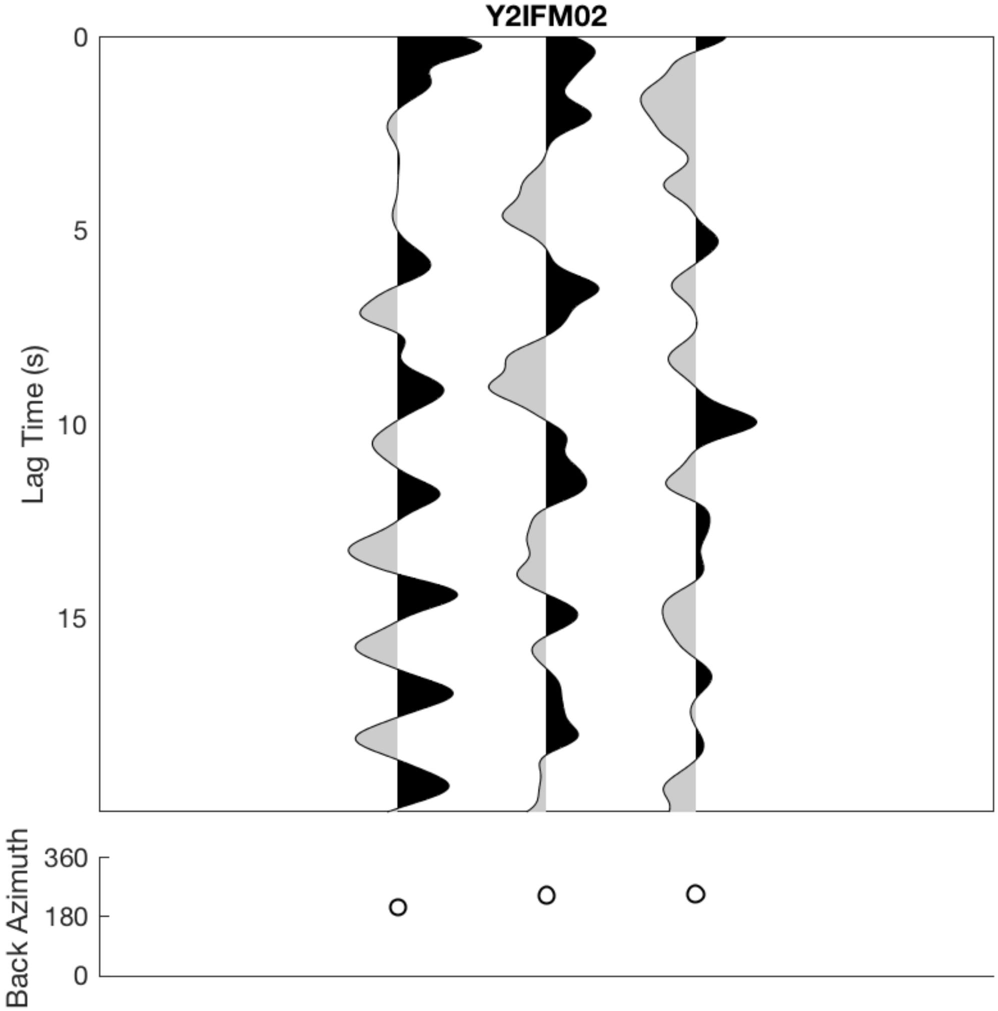

Figure S2: Receiver functions from station Y2IFM02. Format explained in Figure S1.

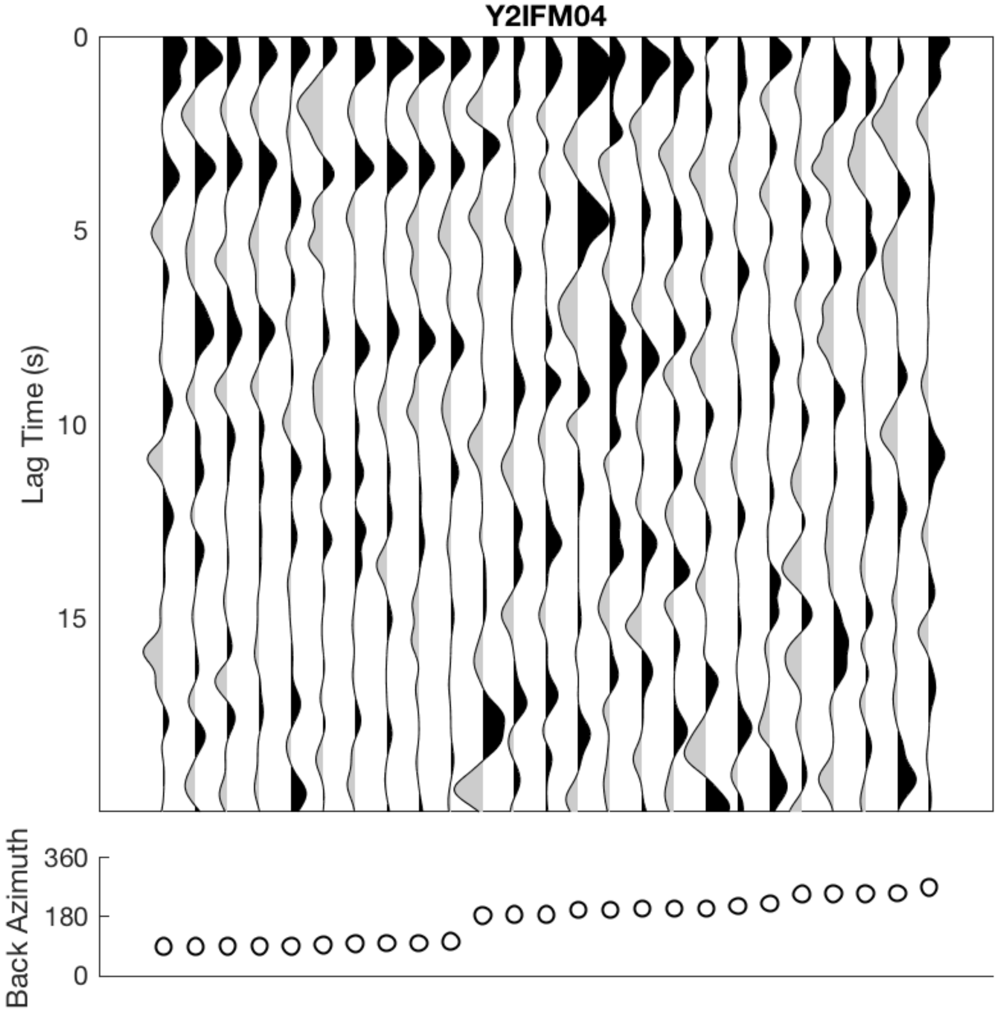

Figure S3: Receiver functions from station Y2IFM04. Format explained in Figure S1.

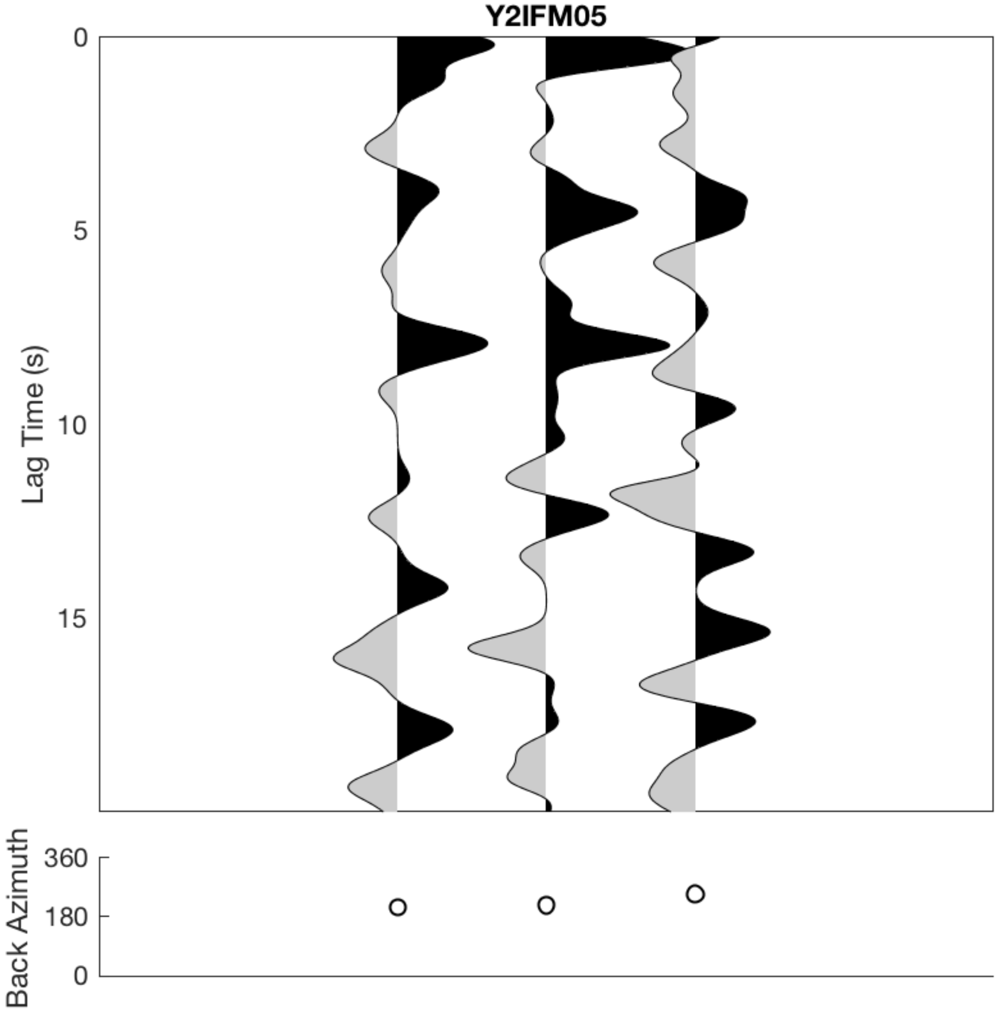

Figure S4: Receiver functions from station Y2IFM05. Format explained in Figure S1.

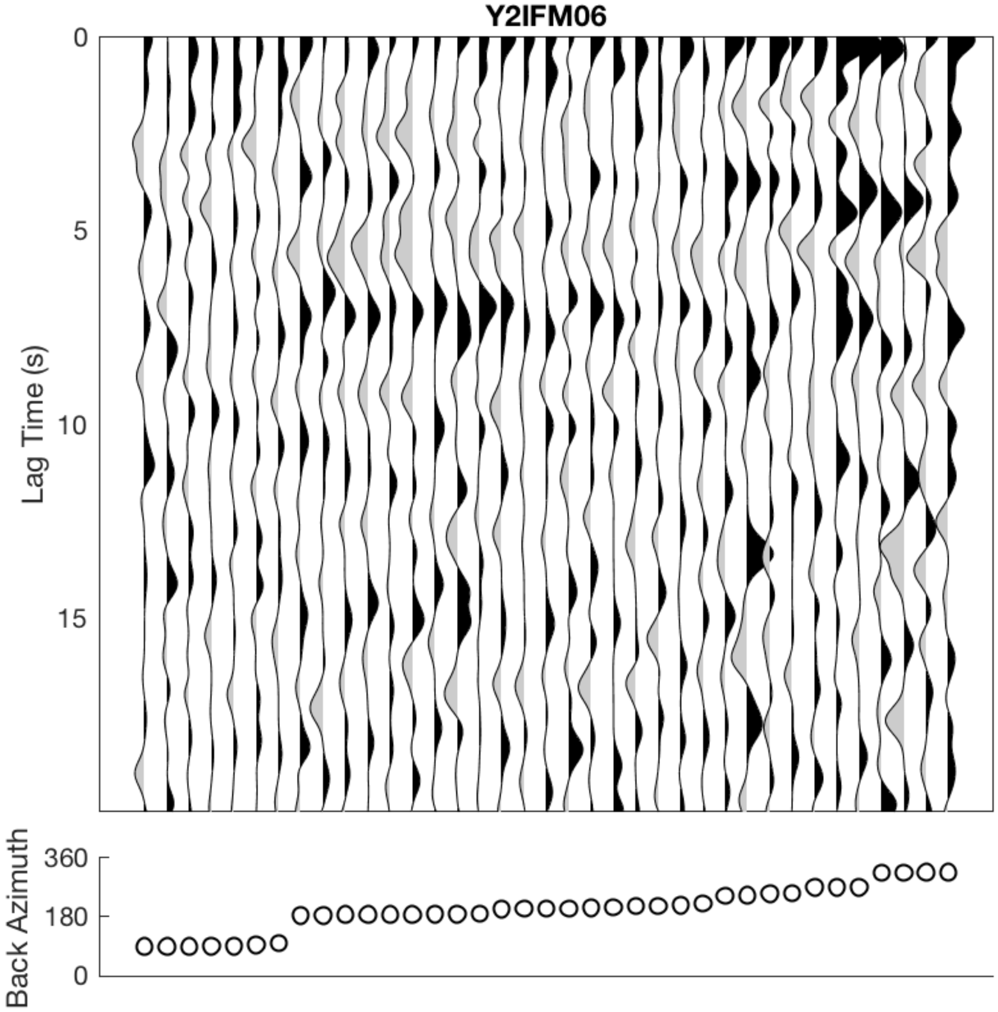

Figure S5: Receiver functions from station Y2IFM06. Format explained in Figure S1.

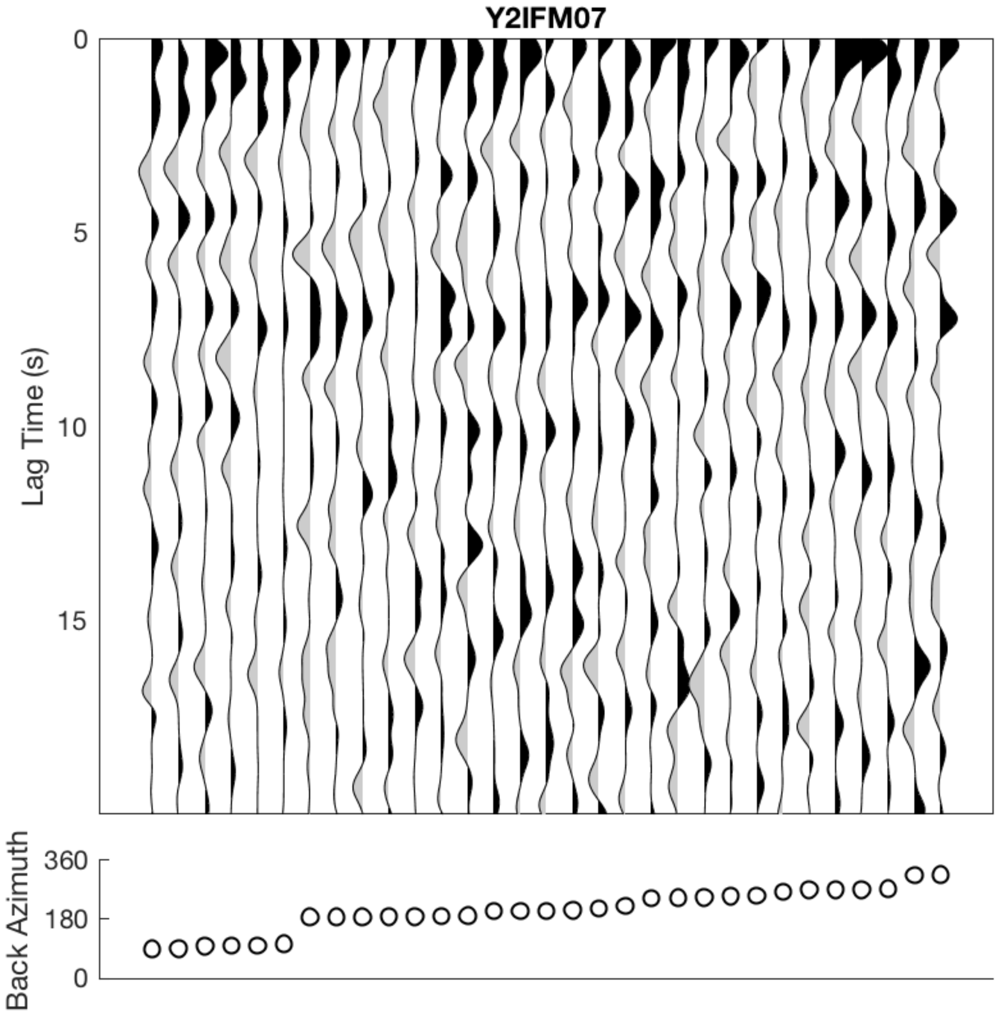

Figure S6: Receiver functions from station Y2IFM07. Format explained in Figure S1.

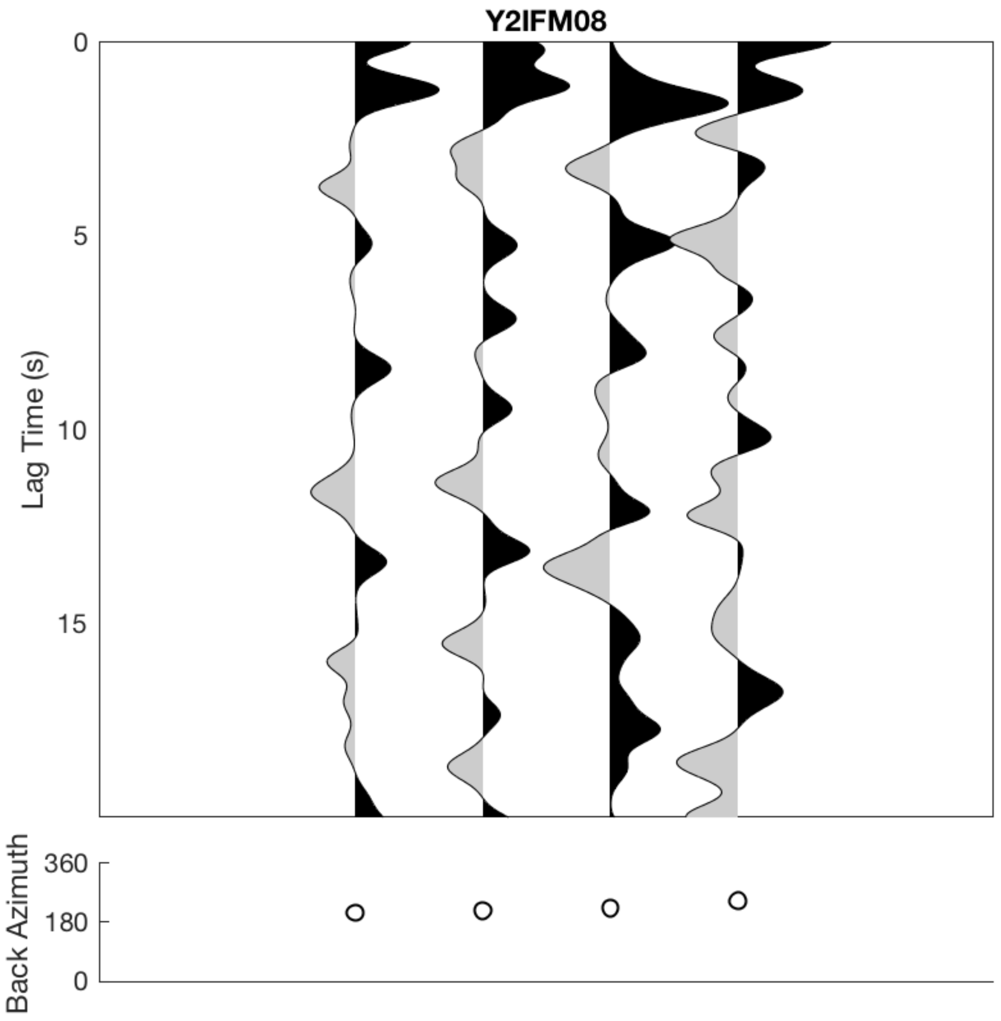

Figure S7: Receiver functions from station Y2IFM08. Format explained in Figure S1.

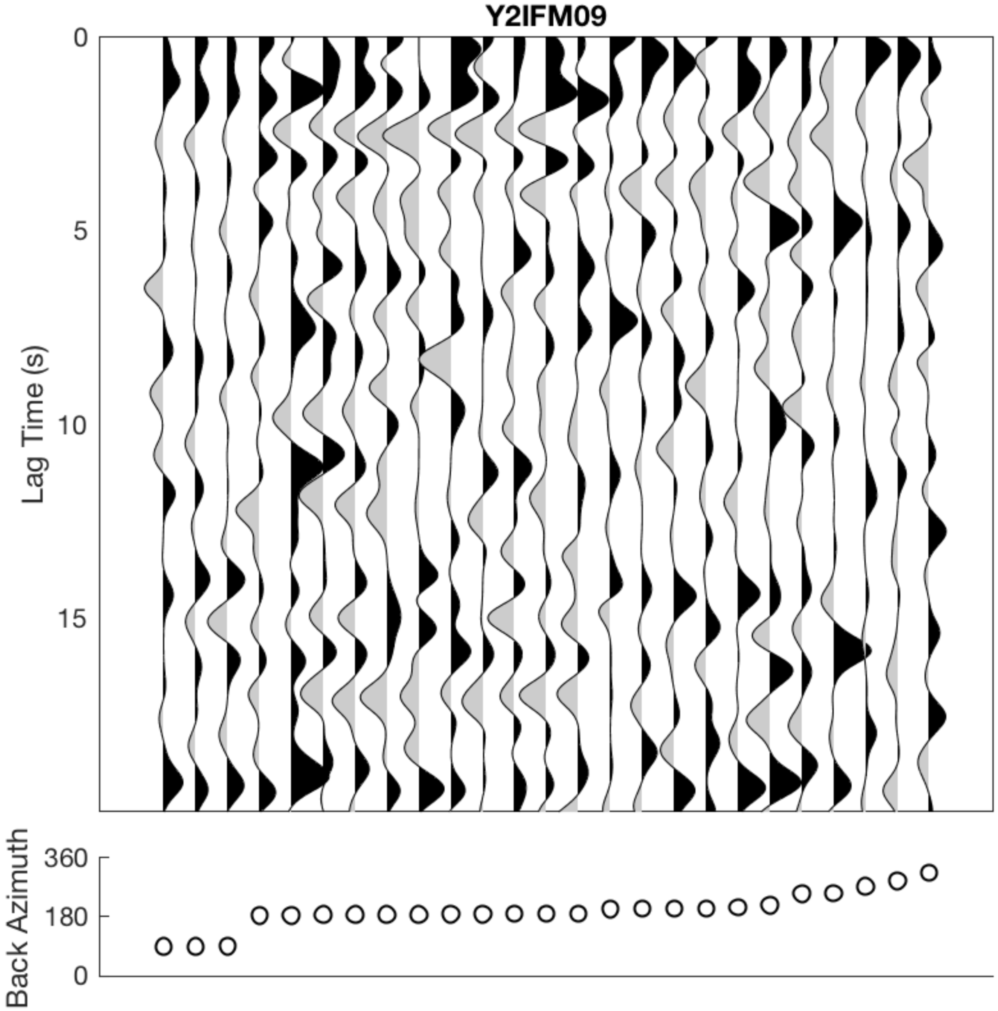

Figure S8: Receiver functions from station Y2IFM09. Format explained in Figure S1.

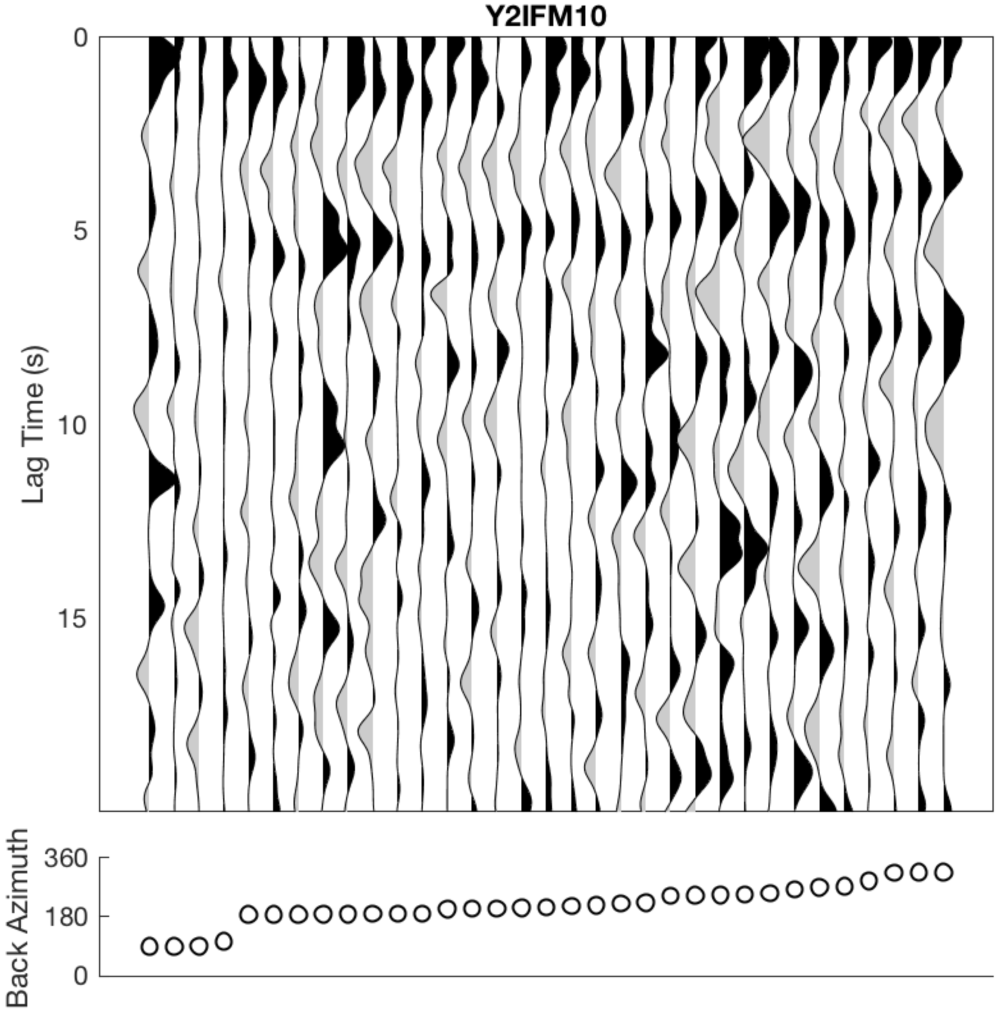

Figure S9: Receiver functions from station Y2IFM10. Format explained in Figure S1.

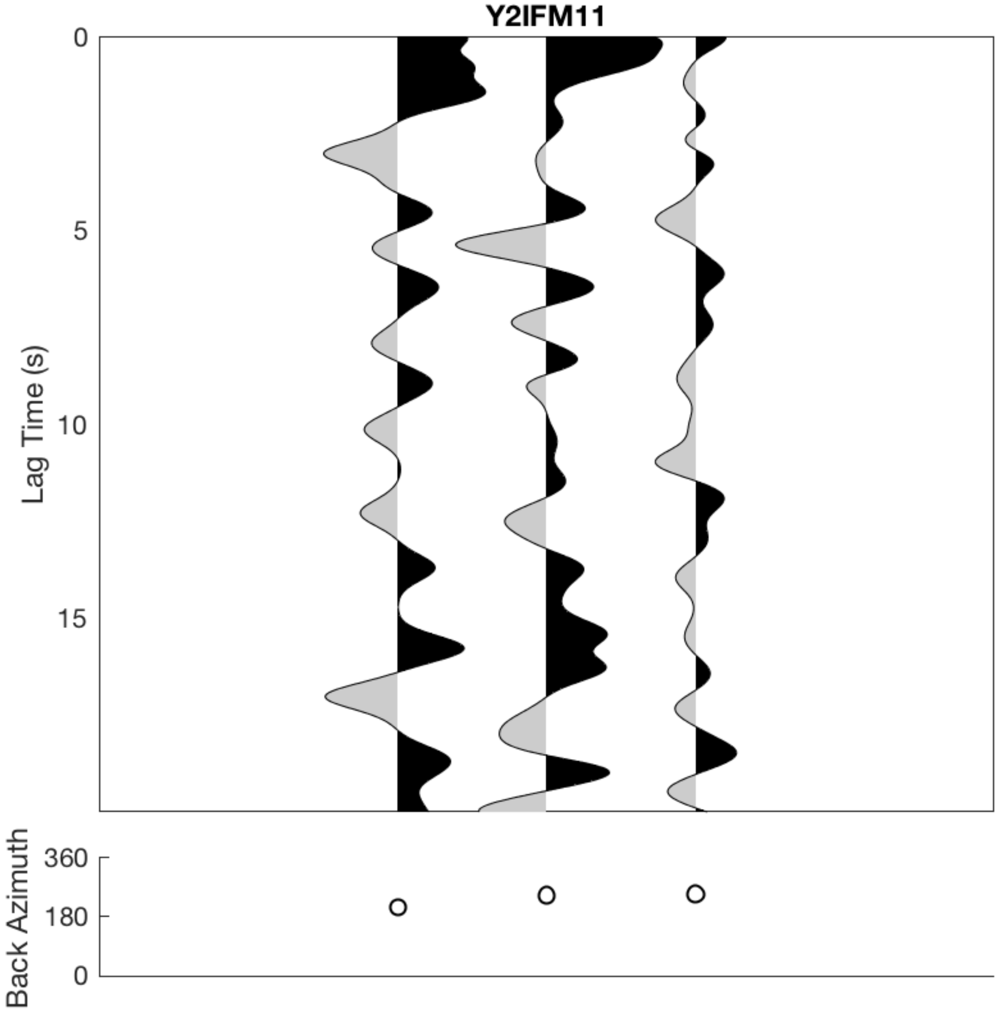

Figure S10: Receiver functions from station Y2IFM11. Format explained in Figure S1.

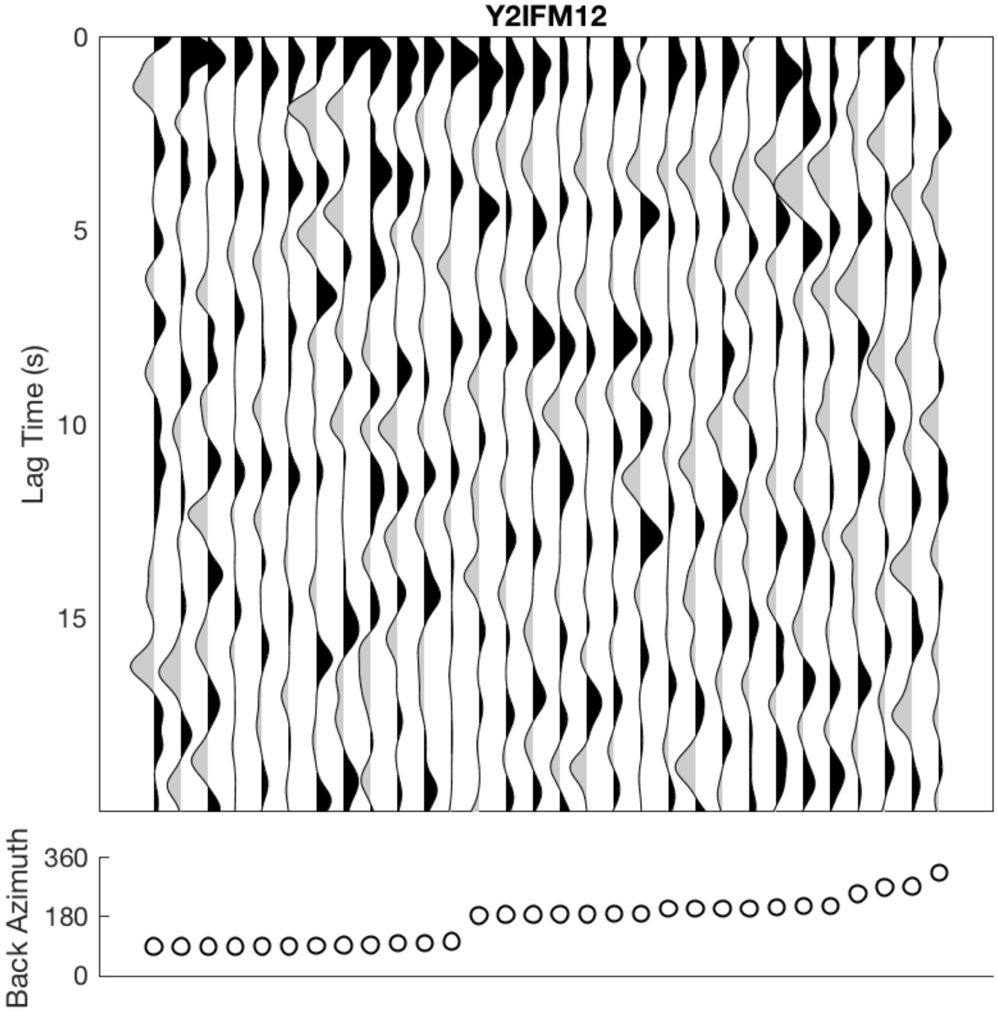

Figure S11: Receiver functions from station Y2IFM12. Format explained in Figure S1.

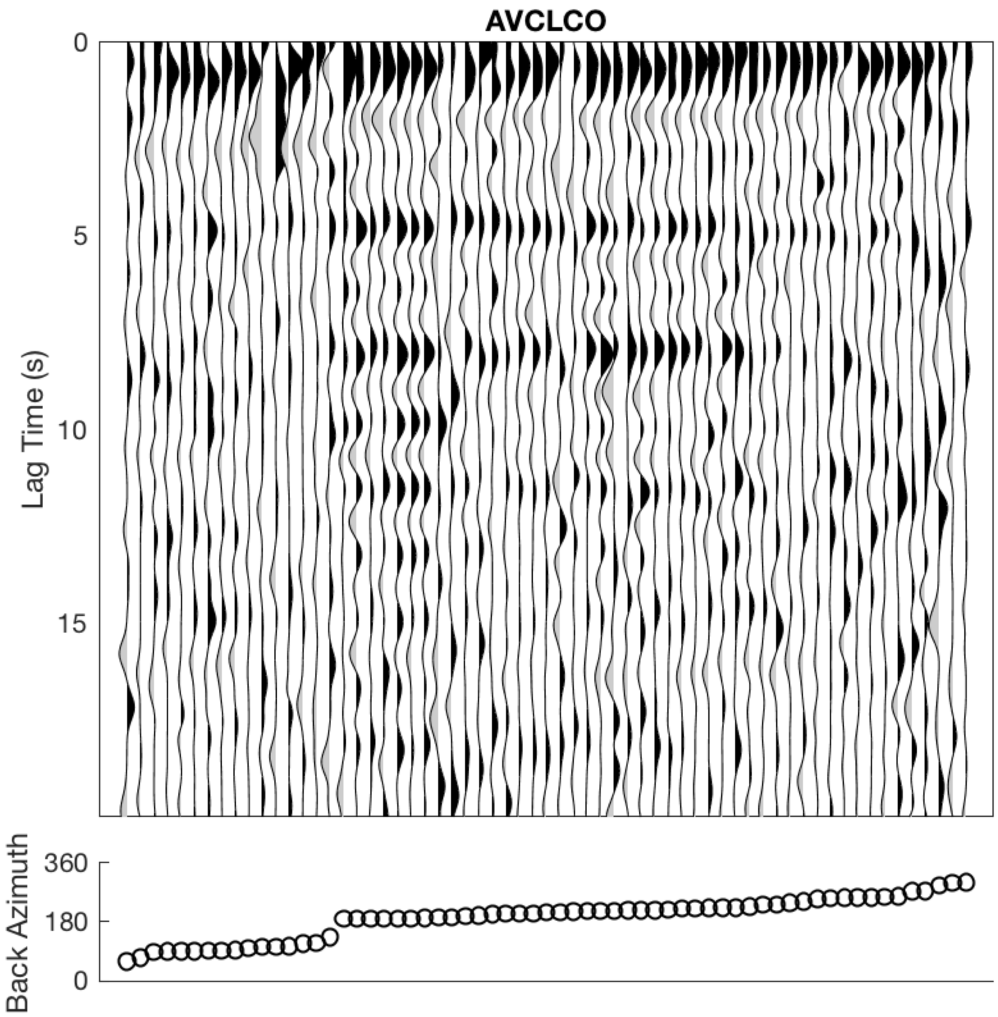

Figure S12: Receiver functions from station AVCLCO. Format explained in Figure S1.

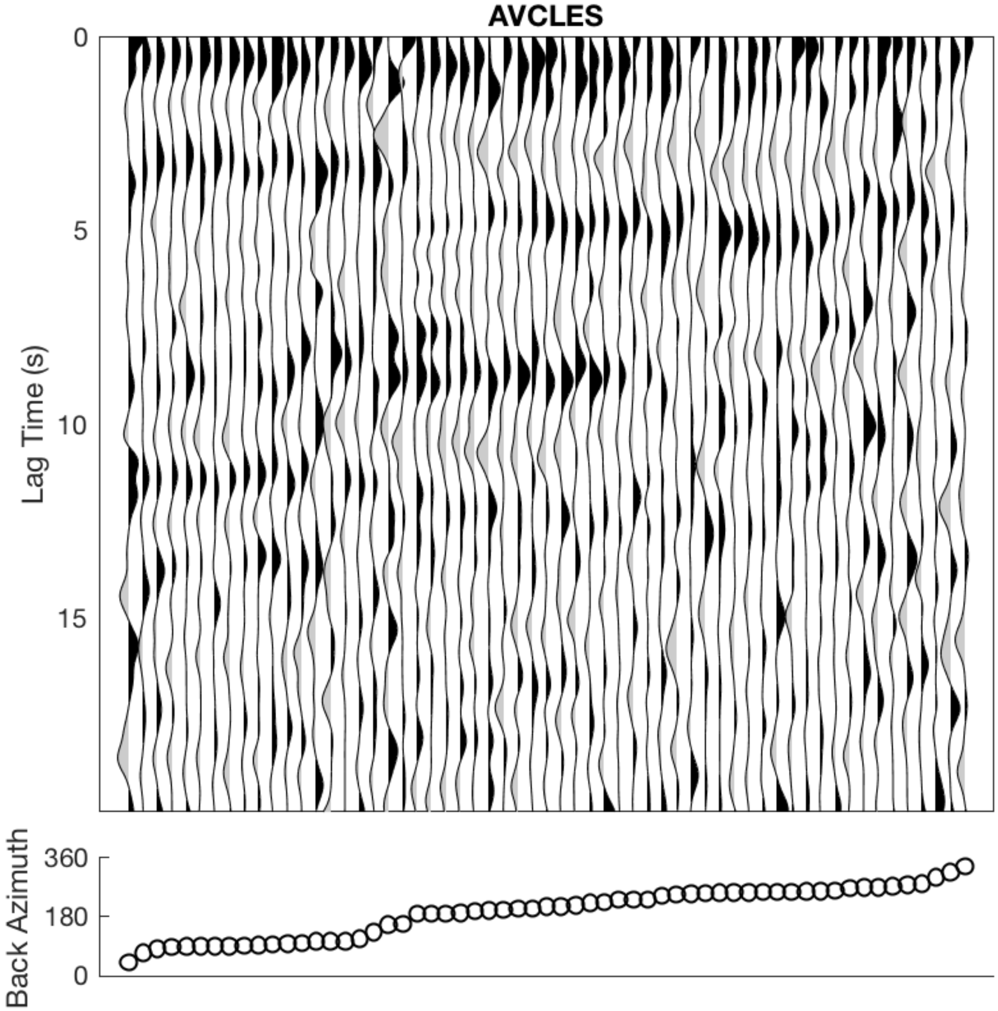

Figure S13: Receiver functions from station AVCLES. Format explained in Figure S1.

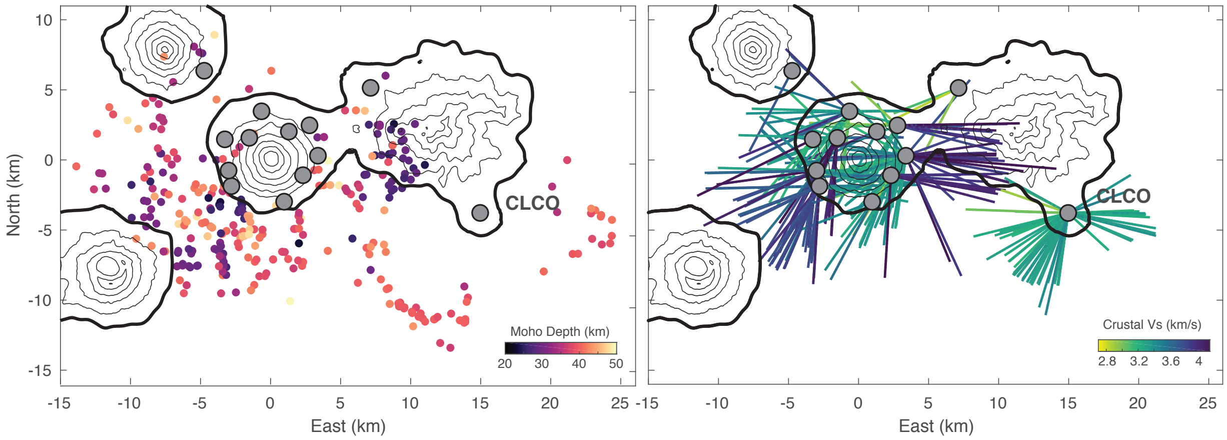

Figure S14: Similar to Figure 3 but including the raypaths from CLCO. These mostly do not intersect with raypaths from the array around the main edifice, indicating that it is possible that systematically different crust may underlie station CLCO.

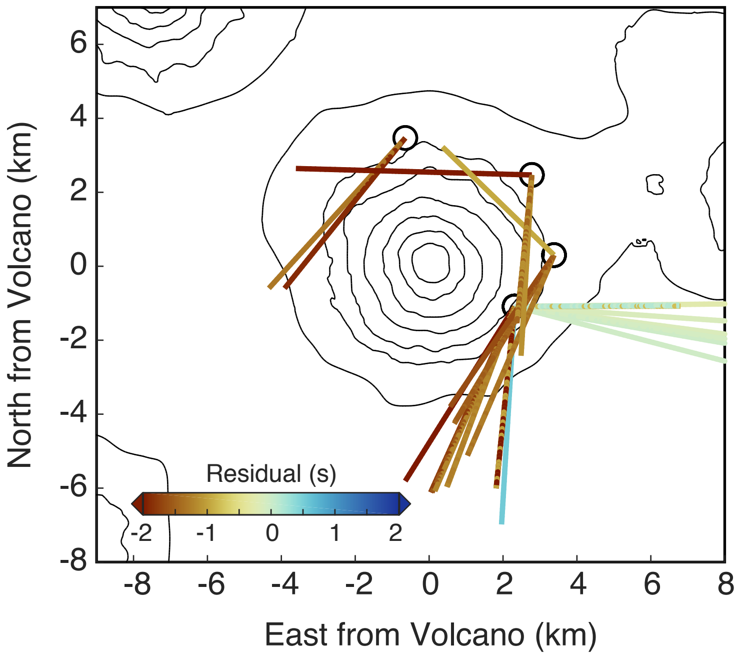

Figure S15: The additional raypaths that pass through the cylindrival LVZ that are present in a 3 km radius model but are absent in a 2.5 km radius model. The colors of the raypaths are the residuals for the baseline model. At the 3 km radius, raypaths that are well fit by the baseline (e.g. that should not pass through a LVZ) are included, indicating that the cylindrical LVZ approximation would not be appropriate.
